# Supplementary material for: Exploring parents’ experiences, attitudes and understanding of gastro-oesophageal reflux in infants
Source: PLoS One. 2024 Sep 23;19(9):e0309081. doi: 10.1371/journal.pone.0309081 (PMC11419391; doi:10.1371/journal.pone.0309081)
Supplement: S1 File — (DOCX) [file pone.0309081.s001.docx]

**Supporting Information File S1: Semi-structured interview guide**

### Title of Study: Exploring parents’ experiences, attitudes and understanding of gastro-oesophageal reflux in infants

**Introduction**:

Ensure participant has read Participant Information Sheet and allow time to answer any questions.

**Purpose of study**: We are interested in hearing about your attitudes and experiences regarding your infant’s gastro-oesophageal reflux (GOR) or gastro-oesophageal reflux disease (GORD) in order to improve understanding and inform future approaches to parental education and support, as well as to inform future research.

Note: Gastro-oesophageal reflux (GOR) occurs when the acid from the stomach travels up into the food pipe (oesophagus) and mouth. Gastro-oesophageal reflux disease (GORD) is when GOR occurs but causes troublesome symptoms.

Reiterate points around confidentiality, data storage, right to withdraw and recording of interview.

**Length**: up to 45 minutes.

Obtain consent (see Consent Form).

**Demographic information**:

M / F

Age

Region of UK

Ethnicity

Highest education qualification

Number of children

**Background information about the infant**:

Age of infant

Month and year of infant’s birth

Term / Preterm

Timing (month and year) of infant GOR/GORD diagnosis

Infant’s age at time of GOR/GORD diagnosis

**Knowledge and understanding**:

1. Please tell me a bit about what you know about infant GOR/GORD.

*Prompts:*

- 1. How common do you think GOR/GORD is in children under 1? *[NICE state nearly half (at least 4 out of 10) of babies under 1 year]*
  2. Can you tell me what the most common symptoms of GOR/GORD are? *[Regurgitation, vomiting, coughing/hiccupping when feeding, unsettled during feeding, crying and not settling, not gaining weight, unsettled during feeding].*
  3. What would you describe as the most troublesome symptoms?
  4. What do you understand about how GOR/GORD is treated or managed? *[if breastfeeding, could do assessment, could try alginate. Bottle fed, smaller more often feeds, adding thickener to milk, then could try alginate. If other symptoms in addition to regurgitation, could try 4 week trial of PPI or H2RA)*
  5. What are the ways in which you have developed this knowledge?

**Diagnosis of GORD:**

1. Please tell me about your experiences of how your infant was diagnosed with GOR/GORD.

*Prompts:*

- 1. When did the symptoms start and what were these?
  2. What were your initial thoughts on what might be causing the symptoms?
     1. At what point did you become concerned that something wasn’t right?
  3. At what point did you think it might be GOR/GORD? And what made you think this?
  4. Which health professionals did you see?
     1. Talk me through what happened?
     2. What did you think about these interactions?
     3. What did they do?
  5. How much time did it take from when you first saw a health professional for a GOR/GORD diagnosis to be reached?
  6. Was there anything else that health professionals thought could be causing the symptoms before the GOR/GORD diagnosis was reached?
  7. What did you feel about health services and health professionals during this time (around diagnosis)?
  8. Were any diagnostic tests done?
  9. How did you feel about the diagnosis?
     1. If so, in what ways has it affected you?

**Management of GORD:**

1. Please tell me about how your infant’s GOR/GORD has been/is being managed?

*Prompts:*

- 1. Is this a medication? If so, please tell me a bit more about that.
  2. How have you found this method?
     1. Did the treatment make any difference?
        1. What changes did you see?
        2. How was your baby or baby’s condition different to how it was before the treatment?
  3. Was anything else trialled before this?
     1. If so, what were your experiences with that method?
  4. How do you feel your child’s GOR/GORD has been managed overall?
     1. Is there anything you would like to have been done differently?
     2. Any particular examples of positive or negative experiences?
  5. Are there any other treatment/management options that you would like to try?
  6. What are your opinions on medical treatment vs at home/conservative management methods?
  7. Are you accessing any form of ongoing support?
  8. Is there any other form of support that you would like?

**Other:**

1. Is there anything that you wish you had known or been told sooner?
   1. Is there any advice that you would give to other parents whose infants is having GOR -like symptoms or who has been recently diagnosed with GOR/GORD?
2. Is there anything else that you would like me to know about your experiences of having an infant with GOR/GORD that we have not covered?
