# Supplementary material for: Exploring parents’ experiences, attitudes and understanding of gastro-oesophageal reflux in infants
Source: PLoS One. 2024 Sep 23;19(9):e0309081. doi: 10.1371/journal.pone.0309081 (PMC11419391; doi:10.1371/journal.pone.0309081)
Supplement: Attachment — Submitted filename: PONE-D-24-01188-WW.pdf [file pone.0309081.s002.pdf]

PLOS ONE

Exploring parents’ experiences, attitudes and understanding of gastro-oesophageal reflux in infants

--Manuscript Draft--

**Manuscript Number:**	PONE-D-24-01188
**Article Type:**	Research Article
**Full Title:**	Exploring parents’ experiences, attitudes and understanding of gastro-oesophageal reflux in infants
**Short Title:**	Parents’ experiences of infant reflux
**Corresponding Author:**	Manpreet Bains, PhD<br>University of Nottingham School of Medicine<br>Nottingham, UNITED KINGDOM
**Keywords:**	gastro-oesophageal reflux, infants, parent experience, qualitative
**Abstract:**	Background: Gastro-oesophageal reflux (GOR) affects nearly half of infants. Parents play a crucial role in management but more understanding of their attitudes and experiences is needed to inform future education, support and research. This study aims to explore parental experiences, attitudes and understanding of the symptoms, diagnosis and management of infant GOR.<br><br>Methods: Qualitative semi-structured interviews with 9 parents of infants with GOR in the UK, analysed by thematic analysis.<br><br>Results: 8 participants were mothers and median age was 34 years. Over half identified as White ethnicity. Parents described that GOR can affect all aspects of life, including mental wellbeing and bonding with their baby. Medications are time-consuming to prepare and can cause challenging side effects such as constipation. It is crucial that health professionals manage parental expectations in that treatments are not curative and symptoms do not last forever. Attitudes about healthcare professionals varied: some were perceived as dismissive, whilst some showed understanding. There were differences depending on whether the child was a first or second born child, with more understanding shown where the child was not the parents’ first born. Parents felt more education could be beneficial for parents and clinicians.<br><br>Conclusions: Infant GOR can affect infants and parents in a variety of ways, impacting both physical and mental health. Parents play a vital role in the management of infant reflux, but there is lack of consistency of information and levels of knowledge among healthcare professionals vary. More education could be beneficial, and further research is needed into health professionals’ perceptions and fathers’ experiences.
**Order of Authors:**	Kathryn McVicar
	Lisa Szatkowski, PhD
	Shalini Ojha
	Simon Tunster
	Manpreet Bains, PhD
**Additional Information:**	
**Question**	**Response**
**Financial Disclosure**<br><br>Enter a financial disclosure statement that describes the sources of funding for the work included in this submission. Review the submission guidelines for detailed	The author(s) received no specific funding for this work.

Powered by Editorial Manager® and ProduXion Manager® from Aries Systems Corporation

requirements. View published research articles from *PLOS ONE* for specific examples.<br><br>This statement is required for submission and **will appear in the published article** if the submission is accepted. Please make sure it is accurate.<br><br>**Funded studies**<br>Enter a statement with the following details:<br>• Initials of the authors who received each award<br>• Grant numbers awarded to each author<br>• The full name of each funder<br>• URL of each funder website<br>• Did the sponsors or funders play any role in the study design, data collection and analysis, decision to publish, or preparation of the manuscript?<br><br>Did you receive funding for this work?	
**Competing Interests**<br><br>Use the instructions below to enter a competing interest statement for this submission. On behalf of all authors, disclose any competing interests that could be perceived to bias this work—acknowledging all financial support and any other relevant financial or non-financial competing interests.<br><br>This statement is required for submission and **will appear in the published article** if the submission is accepted. Please make sure it is accurate and that any funding sources listed in your Funding Information later in the submission form are also declared in your Financial Disclosure statement.<br><br>View published research articles from *PLOS ONE* for specific examples.	The authors have declared that no competing interests exist.

Powered by Editorial Manager® and ProduXion Manager® from Aries Systems Corporation

**NO authors have competing interests**<br><br>Enter: *The authors have declared that no competing interests exist.*<br><br>**Authors with competing interests**<br><br>Enter competing interest details beginning with this statement:<br><br>*I have read the journal's policy and the authors of this manuscript have the following competing interests: [insert competing interests here]*<br><br>* typeset	
**Ethics Statement**<br><br>Enter an ethics statement for this submission. This statement is required if the study involved:<br><br>• Human participants<br>• Human specimens or tissue<br>• Vertebrate animals or cephalopods<br>• Vertebrate embryos or tissues<br>• Field research<br><br>Write "N/A" if the submission does not require an ethics statement.<br><br>General guidance is provided below. Consult the submission guidelines for detailed instructions. **Make sure that all information entered here is included in the Methods section of the manuscript.**	Ethical approval for the study was granted by the University of Nottingham Master of Public Health Ethics Committee on 28th September 2022.

**Format for specific study types**<br><br>**Human Subject Research (involving human participants and/or tissue)**<br>• Give the name of the institutional review board or ethics committee that approved the study<br>• Include the approval number and/or a statement indicating approval of this research<br>• Indicate the form of consent obtained (written/oral) or the reason that consent was not obtained (e.g. the data were analyzed anonymously)<br><br>**Animal Research (involving vertebrate animals, embryos or tissues)**<br>• Provide the name of the Institutional Animal Care and Use Committee (IACUC) or other relevant ethics board that reviewed the study protocol, and indicate whether they approved this research or granted a formal waiver of ethical approval<br>• Include an approval number if one was obtained<br>• If the study involved *non-human primates*, add *additional details* about animal welfare and steps taken to ameliorate suffering<br>• If anesthesia, euthanasia, or any kind of animal sacrifice is part of the study, include briefly which substances and/or methods were applied<br><br>**Field Research**<br><br>Include the following details if this study involves the collection of plant, animal, or other materials from a natural setting:<br>• Field permit number<br>• Name of the institution or relevant body that granted permission	
**Data Availability**<br><br>Authors are required to make all data underlying the findings described fully available, without restriction, and from the time of publication. PLOS allows rare exceptions to address legal and ethical concerns. See the PLOS Data Policy and FAQ for detailed information.	No - some restrictions will apply

Powered by Editorial Manager® and ProduXion Manager® from Aries Systems Corporation

A Data Availability Statement describing where the data can be found is required at submission. Your answers to this question constitute the Data Availability Statement and **will be published in the article**, if accepted.<br><br>**Important:** Stating 'data available on request from the author' is not sufficient. If your data are only available upon request, select 'No' for the first question and explain your exceptional situation in the text box.<br><br>Do the authors confirm that all data underlying the findings described in their manuscript are fully available without restriction?	
**Describe where the data may be found in full sentences. If you are copying our sample text, replace any instances of XXX with the appropriate details.**<br><br>• If the data are **held or will be held in a public repository**, include URLs, accession numbers or DOIs. If this information will only be available after acceptance, indicate this by ticking the box below. For example: *All XXX files are available from the XXX database (accession number(s) XXX, XXX.).*<br>• If the data are all contained **within the manuscript and/or Supporting Information files**, enter the following: *All relevant data are within the manuscript and its Supporting Information files.*<br>• If neither of these applies but you are able to provide **details of access elsewhere**, with or without limitations, please do so. For example:<br><br>*Data cannot be shared publicly because of [XXX]. Data are available from the XXX Institutional Data Access / Ethics Committee (contact via XXX) for researchers who meet the criteria for access to confidential data.*<br><br>*The data underlying the results presented in the study are available from (include the name of the third party*	The data underlying the results presented in the study are available from the authors, upon request, for researchers who meet the criteria for access to confidential data.

and contact information or URL).<br>• This text is appropriate if the data are owned by a third party and authors do not have permission to share the data.<br><br>* typeset	
Additional data availability information:	

Click here to access/download;Manuscript;McVicar et al. Parents experiences of infant reflux.docx

Exploring parents' experiences, attitudes and understanding of gastro-oesophageal reflux in infants

Short title: Parents' experiences of infant reflux

Kathryn McVicar[1], Lisa Szatkowski[1], Shalini Ojha[1,2], Simon Tunster[1], Manpreet Bains[1]

[1] Lifespan and Population Health, School of Medicine, University of Nottingham, UK

[2] Neonatal Unit, University Hospitals of Derby and Burton NHS Trust, Derby, UK

Corresponding author: Dr Manpreet Bains, Lifespan and Population Health, School of Medicine, University of Nottingham, UK. Email: manpreet.bains@nottingham.ac.uk

Abstract

Background: Gastro-oesophageal reflux (GOR) affects nearly half of infants. Parents play a crucial role in management but more understanding of their attitudes and experiences is needed to inform future education, support and research. This study aims to explore parental experiences, attitudes and understanding of the symptoms, diagnosis and management of infant GOR.

Methods: Qualitative semi-structured interviews with 9 parents of infants with GOR in the UK, analysed by thematic analysis.

Results: 8 participants were mothers and median age was 34 years. Over half identified as White ethnicity. Parents described that GOR can affect all aspects of life, including mental wellbeing and bonding with their baby. Medications are time-consuming to prepare and can cause challenging side effects such as constipation. It is crucial that health professionals manage parental expectations in that treatments are not curative and symptoms do not last forever. Attitudes about healthcare professionals varied: some were perceived as dismissive, whilst some showed understanding. There were differences depending on whether the child was a first or second born child, with more understanding shown where the child was not the parents' first born. Parents felt more education could be beneficial for parents and clinicians.

Conclusions: Infant GOR can affect infants and parents in a variety of ways, impacting both physical and mental health. Parents play a vital role in the management of infant reflux, but there is lack of consistency of information and levels of knowledge among healthcare professionals vary. More education could be beneficial, and further research is needed into health professionals' perceptions and fathers' experiences.

Introduction

Gastro-oesophageal reflux (GOR) is defined as ‘the involuntary retrograde passage of gastric contents into the oesophagus with or without regurgitation’[1]. It is thought to affect just under half of infants (children aged under one year) at least once a day because the ring of muscle at the base of the oesophagus has not yet matured[1–3]. Reflux is especially common in preterm infants (born ≤37 weeks’ gestation) but affects infants born both preterm and at term. Symptoms usually start before a child is eight weeks old and normally improve with age[2]. When infants do not experience severe complications and continue to thrive, GOR is considered physiological and is usually self-limiting[2,4]. However, reflux can occur with increasing frequency and severity which can damage the oesophageal mucosa and can cause pathological (or ‘troublesome’) symptoms or complications[1,2,4]. This is known as gastro-oesophageal reflux disease (GORD)[2]. GOR and GORD are often seen on a continuum, and it is often difficult to distinguish between them given the unclear boundary between physiological and pathological symptoms[1,5].

Diagnosis of GOR or GORD in infants is most commonly based solely on the clinical history provided by the parent or carer[3]. The most common sign is frequent spitting up or regurgitation after feeds, but signs can be non-specific and often overlap with ‘normal’ infant behaviour, such as excessive crying or distress[3,5]. This can make reflux difficult to diagnose and manage, and the condition has been described as ‘commonly misunderstood’[6]. ~~Research has shown that~~ crying infants are often overlabeled as having the condition and overprescribing of medications may be occuring[7–9]. The use of the label ‘GORD’ itself can sometimes affect parental decisions and expectations regarding pharmacological treatment[10].

There is a lack of clear, evidence-based guidelines for diagnosis and management of GOR/GORD[9,11,12], potentially due to the lack of gold standard objective measures and

reliance on the reporting of signs and symptoms[1]. The National Institute of Health and Care Excellence (NICE) guidance for the management of GOR/GORD in infants recommends parental reassurance and education on conservative management (such as keeping an infant upright after feeds and offering smaller, more frequent feeds) as first-line options[2]. A number of studies have shown the centrality of parents in the diagnosis and management of GOR/GORD[5,6,13].

Having an infant with GOR/GORD has been shown to be distressing to parents and can affect parental wellbeing, leading to high levels of anxiety[14]. Infant irritability is a common symptom of GOR/GORD and this has been linked to maternal fatigue, anxiety and depression[15]. Additionally it could negatively impact bonding between the infant and parent during feeding, with mothers of infants with GOR/GORD experiencing worse interactions with their child than those without[5,16].

Much of the research into infant GOR/GORD and the role of parents in the diagnosis and management has not been conducted in the United Kingdom, but other nations have identified that a greater understanding of parents knowledge of, and attitudes towards the condition is needed to further understand the pressures on parents[5,6,9]. The purpose of this study is to explore parental experiences, attitudes and understanding of the symptoms, diagnosis and management of infant GOR and GORD. This could help inform future research, as well as future approaches to parental education and support, which is fundamental to GOR/GORD management[17].

Methods

This was a qualitative study involving semi-structured interviews with parents in the United Kingdom of children with GOR/GORD. A constructionist approach was taken, which assumes that meaning and experience are socially constructed[18,19]. This allowed us to gain

understanding of participants' experiences, recognising that findings would be co-constructed by the researchers and participants[20].

Ethical approval for the study was granted by the University of Nottingham Master of Public Health Ethics Committee on 28th September 2022.

Study population, sampling and recruitment

A combination of convenience (self-selecting) and purposive sampling approaches were used to recruit a range of parents including those with infants born preterm and at term. Participants were recruited between 01 December 2022 and 17 May 2023 via adverts posted on the online forums of Mumsnet and Facebook, a poster at a children's playgroup in Nottinghamshire, and through adverts circulated through local parents' WhatsApp groups. Participants were eligible if they lived in the UK, were aged 18 or older, able to complete the interview in English, and were a parent or guardian of a child aged under 2 years at the time of interview who was diagnosed with GOR/GORD before their first birthday.

Interview guide and procedure

The semi-structured interview guide (see Supplementary File 1) was developed based on the research aims, existing literature and NICE guidance[2]. The main topics covered were knowledge and understanding of infant GOR/GORD, diagnosis experiences, and experiences and attitudes about the management of GOR/GORD.

Prior to the interview, participants were sent a Participant Information Sheet, given the opportunity to ask questions, and completed a consent form via Microsoft Forms. At the start of each interview the researcher reiterated points about withdrawal from the study and data confidentiality, verbally confirmed consent and checked the participant was still happy to proceed.

Interviews were completed by telephone or online (primarily Microsoft Teams) depending on participant preference and were recorded. A short demographic questionnaire was completed at the start of the interview. Participants received a £15 voucher for participation. The first interview served as a pilot resulting in amendment of one question to improve clarity.

Data analysis

Interviews were transcribed verbatim by KM and transcripts were checked to ensure accuracy and to anonymise any personally identifiable data. Each participant was assigned a unique code which was ascribed to the corresponding transcript and audio file.

Data was analysed thematically using an inductive approach which allowed meaning to be generated from the data[21,22]. Firstly, familiarity was developed through re-reading the transcripts, where initial reflections were documented. Next, NVivo[23] was used to facilitate axial and analytical coding[24]. These initial stages facilitated interpretations at the semantic level[25].

Themes were then formed by collating codes with similar characteristics. At this stage analysis moved to a latent level[21]. Thematic maps were developed using NVivo to organise themes and corresponding subthemes. A small number of transcripts were independently double-coded by MB and ST to enhance validity[26]. Themes were reviewed using an iterative approach, where they were adapted until they accurately represented the data.

Results

Participants

Between December 2022 and May 2023, twelve individuals expressed interest in participating; nine were interviewed and three were unreachable. Interviews averaged 32 minutes (ranging between 18 and 46 minutes).

The median age of participants was 34 years (interquartile range, IQR, 30-36 years) and the median age of their child was 8 months (IQR 4-17 months). All but one of the participants were the child's mother and one was the child's father. Five were White British, three identified as White Other and one was Black British. All nine participants were educated to at least A-level equivalent, seven to at least Bachelor's degree level and four to Master's degree level. Five participants had one child; the remainder had two. Five of the participants' infants were born at term and four preterm.

All but one of the participants' infants were diagnosed with GOR/GORD within the first eight weeks of life (median 4 weeks, IQR 2-7). Symptoms commonly described by parents were regurgitation of milk, discomfort, crying, breathing difficulties and difficulty gaining or maintaining weight. All participants understood that reflux occurs due to passage of gastric contents into the oesophagus, and 5 also demonstrated knowledge and/or experience of silent reflux, where regurgitation of milk is not visible. All had tried conservative (or home-management) methods, such as smaller more frequent feeds, as well as some form of medical management for their infant's reflux; for two this was milk thickener only, seven had tried Gaviscon and six had also tried omeprazole.

Overview of themes

Four themes with corresponding sub-themes were identified (Table 1). Each theme is outlined below with supporting quotes labelled with the participant identifier. No further identifying details are given to preserve anonymity.

Table 1: Themes and sub-themes

Themes	Subthemes
The impact of GOR/GORD on all aspects of family life	- Impact on activities of daily living, mental health and the role of support

	- Reality of parenting not matching expectations
The challenges in the management of GOR/GORD in infants	- Struggles with different medications<br>- Importance of managing expectations
The role of healthcare professionals in the diagnosis and management of GOR/GORD	- Importance of the attitudes of health professionals<br>- Overlap of healthcare professional role with parental role<br>- Lack of continuity of care
The need for more information and education on the symptoms and management of GOR/GORD	- For clinicians<br>- For parents and parents-to-be

The impact of GOR/GORD on all aspects of family life

Impact on activities of daily living, mental health and the role of support

Many parents described the challenges that having an infant with reflux adds to day-to-day life, such as leaving the house, seeing friends and family, and going to the toilet. Several parents described that they were unable to lay the child down as this was often when reflux symptoms were worst, resulting in them being unable to manage other tasks during the day, or affecting sleep at night, which caused stress.

> *"It was very stressful because, you know, sometimes you might just want, 'oh, I just need to go to the bathroom' and especially when my husband went back to work and, like, I'll just put you down and you know, he starts screaming because he's so uncomfortable, and it's like 'oh God', you know, just things like that. And so I did learn how to do a lot of things one handed." (#5)*

Some parents described the toll that having an infant with reflux can have on mental health and wellbeing. Feelings of helplessness and self-blame were noted, and a few parents were emotional when discussing this, indicating the extent to which this experience affected them.

> *"It would be very easy for your mental health to go really like really downhill, you know, you just had a baby, you're not going anywhere, you're not enjoying looking after them because they're miserable." (#1)*

Parents mentioned the importance of having support, for example spousal support, and one parent indicated that a coffee morning with other parents who are going through the same experience 'would help mental health with parents astronomically.

> *"So like supporting each other is very essential for mental well-being. I say this because on my own, I think if not for my [partner's] strength, I think the illness would have tortured me mentally a bit, but my [partner] was really strong." (#6)*

Reality of parenting not matching expectations

Parents explained the impact that infant GOR/GORD had had on their parenting experiences, and how consequently this had not matched up to their expectations. They felt their lived experiences, such as baby 'crying' or 'screaming' had adversely affected their ability to bond with their child, which mothers reported was more profound for their partners.

> *"I suppose the other notable thing is that my husband has found it really difficult… there was a period of time where [child's name] just cried or actually screamed every*

time [father's name] held him… And [father's name] found it quite difficult to bond with him." (#2)

Many mothers spoke about their breastfeeding experiences, and the particular challenge of having to express milk to be able to administer medications such as Gaviscon and omeprazole (which need to be mixed with milk).

"It was harder because we had to express the milk and put the Gaviscon powders in and then measure everything together and he was still spitting it out." (#9)

Some of these negative experiences impacted decisions about feeding for subsequent children.

"One of the reasons I haven't breast fed the second time round is because it was not a pleasant experience all round… things like giving him the Gaviscon meant that you either give a bit of formula before the feed or express the milk with the Gaviscon before then feeding him, so it was essentially having to breastfeed him and have a bottle… it was a real nightmare." (#1)

The challenges in the management of GOR/GORD in infants

Struggles with different medications

All parents experienced similar challenges with medical treatments for GOR/GORD, such as the time-consuming nature of preparation and administration. While some parents felt that Gaviscon improved symptoms, they were met with constipation, which added another layer

of complexity. However, others felt it made their child worse by seeming to cause more discomfort and distress.

> *"We were kind of going back and forth with [Gaviscon], cause we were trying to stop him sicking up, but then we were struggling because he couldn't push everything through… he was getting extremely constipated and he would be waking up screaming." (#5)*

Six parents were eventually prescribed omeprazole after trialling Gaviscon, with most describing this leading to the biggest improvement in symptoms. However, some had challenges with accessing the different formulations prescribed, when paediatricians had prescribed preparations that are not routine for infants and pharmacies were reluctant to dispense.

> *"The omeprazole really was a game changer … [but] I've had a couple of occasions where the omeprazole capsule has been prescribed and the pharmacy has refused to give it to me because they've said you can't give this capsule to a one year old baby. And I've said 'this is how I'm gonna do it and this is what the paediatrician has told to do' but I've had a pharmacy twice refused to give me the actual prescription because they don't feel comfortable giving it to a baby, despite the fact that the paediatrician had prescribed it for her. So that's been frustrating." (#8)*

Importance of managing expectations

Most parents explained that, given the challenges in managing infant GOR/GORD, managing expectations is vital. Many described the importance of understanding the treatment options are not necessarily a cure and knowing that GOR/GORD does not last forever.

> *"The most helpful thing for me and for us probably is the consultant being really honest about kind of managing our expectations… he was just quite honest and said, you know "he's a reflux-y baby, he will grow out of it, but the next few months are going to be really tough until he does." (#2)*

The role of healthcare professionals in the diagnosis and management of GOR/GORD

Importance of the attitudes of health professionals

Parents described that they encountered different attitudes from health professionals towards GOR/GORD, for example some felt these were patronising and dismissive, which they perceived led to delays in diagnosis or inappropriate management. Conversely, some parents felt they were shown understanding, in particular from health visitors. Health professionals' practices also seemed to be influenced by their own beliefs towards medications for reflux, with some eager to prescribe immediately and some much more reluctant; sometimes this was also felt to be influenced by length of appointment, with medications seen as a 'quick fix'.

> *"For my first when I went to see the doctors talk about it, it was very much like "oh is this your first, you know the babies do cry you know, they do get a bit unhappy and they do have a bit of a bit of sick", so you know, it was patronising… the doctors [didn't] believe me… it must have been between five and six months when we actually got some medication for it." (#1)*

“It was kind of overlooked by a lot of the health professionals at the beginning, just kind of calling it as ‘babies will be babies, you know, babies cry, babies scream, babies arch their backs’, so he was overlooked at the beginning by a lot of the health professionals.” (#3)

“With Gaviscon we were told in the hospital that they didn't want to medicate him. Gaviscon they said had bad side effects in babies.” (#5)

From parents of two children who both experienced GOR/GORD, strong differences in attitudes were noted between their first and second child, with more understanding shown towards the second child.

“With my daughter, though, because of the history with my son, they just listened to me right away.” (#3)

Overlap of healthcare professional role with parental role

Parents explained that they felt most care was driven by them, rather than by health professionals and that this seemed to be because they felt they had become ‘expert’ in the condition. Reading about the symptoms and their experiences online, and through talking to others, aided parents to lead conversations with clinicians and push for a diagnosis.

“Just independent research I think, because like my GP’s are lovely, they've been so great, they've been so helpful. But I wasn't satisfied with just ‘OK, here, put them on some medication’, you know, because medication helps the symptoms and it helps to

relieve the like problem at the time, but long term, what's it going to do? So that's what prompted me to kind of look into reflux more as a whole." (#3)

Lack of continuity of care

Specifically for preterm infants where multiple health professionals were involved in the child's care, parents felt that there was at times a lack of continuity and unclear roles and responsibilities for the management of GOR/GORD. This led to parents feeling that they did not have support available to them when they needed it.

> *"I have found the GP really frustrating because they are always like 'it's a neonatal baby, I don't want to get involved, you need to see the paediatrician', but then I won't help you get an appointment to see the paediatrician…My health visitor wasn't great, I have to be honest. I think she kind of, again, was a bit like… 'well, you're under so many other people that you kind of like don't really need me', but there were times where I actually really did need some help and support, but well, there was no one giving it to me." (#8)*

The need for more information and education on the symptoms and management of GOR/GORD

For clinicians

Many parents felt that there was a need for health professionals to have more training on infant GOR/GORD, particularly regarding distinctions between different diagnoses in infancy (such as cow's milk protein allergy, CMPA) and also related to evidence-based management. Some referred to the need for a clear definition or diagnostic criteria for infant reflux.

> *"I feel as though there needs to be kind of more education on reflux, on underlying causes of it, on how to treat it, than just what's already available to our medical professionals. I don't know if you know, maybe some further like CPD or training could go and work wonders, and you know more research could work wonders. Because it did feel like, you know, I had to kind of go off my own back and, you know, take things into my own hands to try and get to the bottom of what was causing the reflux in both of my children."* (#3)

> *"It maybe would be helpful to have a better definition of GOR. Then it would be helpful, maybe for healthcare professionals as well as for parents to say like this is a normal amount of regurgitation and this isn't and kind of needs attention or is troublesome."* (#2)

For parents and parents-to-be

Participants described that, in general, infant reflux is often misunderstood, particularly in relation to the amount of distress it can cause and the ways it impacts those affected, and therefore there could be utility in educating all parents-to-be.

> *"Perhaps sensitising people on reflux, like even before they get children. Like maybe like maybe putting posters in hospitals, mostly in maternity wards or the maternity areas and yeah maybe using newspapers and social media to sensitise people because I realised that I wasn't the only one who didn't know about it."* (#6)

There was also a need for education about GOR/GORD for parents with an affected infant, not only to help with management but also with managing expectations of symptoms and duration. Parents explained that sometimes knowledge was assumed (particularly by General Practitioners and health visitors), and that it would be useful to have more education on the different ways GOR/GORD can present, for example without visible regurgitation.

It was explained that it would be useful to have a reliable source of information, given that there is often mixed and conflicting information or advice. This is both on the internet through lack of credible online sources (or difficulty identifying sources which are credible), and from healthcare professionals (e.g., some suggesting to eliminate dairy, but others stating there is no evidence behind this).

Four parents had professional backgrounds which helped inform their understanding of reflux, but they recognised reliance on this is not an option for all parents. One explained that their professional training did not reflect their personal experience.

> *"It would have been nice to have a bit more information. It was kind of just like he has reflux, there you go, bye. Whereas it would have been nice if there was like, I don't know, just like an information sheet or something. So you have one source of information, you don't need to like go Googling and try and find everything out, they just say 'here's what you do for reflux' or 'these are your options' … My understanding was a little bit different in terms of my training versus in real life experiencing it. It's been a lot different than I kind of was told that it is."* (#7)

Discussion

Study participants demonstrated good knowledge and understanding of the mechanism by which GOR/GORD occurs and its common symptoms, such as regurgitation and discomfort. There were differences between first-time parents and those with more than one child,

indicating that knowledge and experiences obtained from having a child with GOR/GORD leads to increased trust from health professionals or perhaps the ability to frame the situation in a way that better aids support.

Infant GOR/GORD can affect all aspects of life for the family, which can have a significant impact on parental wellbeing. Difficulties with preparation and administration of medications, which are often perceived as ineffective, adds to the burden, particularly amongst breastfeeding mothers. Care was felt to be primarily driven by parents, and parents described that they took on a similar role to health professionals by becoming an expert in GOR/GORD through independent research.

The study indicates how extensively infant GOR/GORD can affect not only the symptomatic child but also the parents. Parents are often unable to carry out their normal activities, which can affect parental mental health and wellbeing. Previous studies have aligned with these findings, demonstrating that infant crying, sleep disturbance and feeding difficulties are associated with higher levels of parenting stress, maternal anxiety and depression[27–29]. However, most prior research focuses on the impact on the mother, whereas in this study mothers and the one father who participated indicated that reflux can also affect the mental wellbeing of fathers.

It is well recognised that feeding is an important bonding experience between parents and babies[30,31]. Breastfeeding in particular is acknowledged as a vehicle to facilitate bonding[31,32]. A small pilot study found that parental-infant interactions were significantly worse where infants had GORD than when they did not[16]. This study echoes these findings, with particular reference to challenges administering medications impacting feeding. It also reveals a novel finding that GOR/GORD can impact a mother’s decision to breast feed future children, an additional factor that could affect parental mental wellbeing[33].

The majority of participants had trialled some form of medical management for infant GOR/GORD, in contrast to published literature and guidance which state for most cases

reassurance alone can suffice. This perhaps reflects prior findings that overprescribing might be occurring, although the self-selective nature of recruitment could have meant that participants' children were more likely to have GORD than GOR, and therefore warranted medical treatment[1,4,9,34,35]. Research has shown that once infants are labelled with having a 'disease' such as GORD, parents are more interested in using medication[10]. The parent-driven nature of reflux management could have therefore played a role as to why most participants had experience with medical treatments.

Participants in this study had mixed experiences with medical management methods. A recent Cochrane review concluded that there is very low certainty evidence regarding whether medications provide benefit in infants where conservative reflux management has proved ineffective[36]. This highlights the importance of ensuring parental expectations are managed. Parents explained the importance health professionals explaining that medications are not curative and providing reassurance that symptoms will not last forever.

This study indicates a lack of consistency in the knowledge and attitudes among healthcare professionals regarding infant GOR/GORD. This could imply that it is not well-taught during training, or could echo findings which emphasise the need for clearer guidelines for diagnosis and management[11,12]. The marked difference in attitudes from professionals between first-born and subsequent children has not been previously identified in this context. This could reflect the increased parental knowledge and experience of having a previous child with GOR/GORD, and links with the parent-driven nature of management. Parents who have experienced GOR/GORD before could know the 'right' things to say to feel heard and understood. Alternatively, health professionals could be more trusting that parents know what they are talking about. This area warrants further exploration.

Existing literature describes that continuity of care can be challenging and multi-layered, especially for neonates transitioning home from intensive care units[37,38]. This was evident in parents of preterm infants, where participants felt that they were dismissed by other health professionals such as General Practitioners if they were under the continued care of

paediatric or neonatal teams. A New Zealand study found that having a designated case manager could aid seamless multi-disciplinary care[38], which could be considered in the UK.

Participants described difficulties with validity and reliability of information on GOR/GORD available on the internet, and particularly the challenges in appraising the quality of evidence. This is a well-recognised and long standing issue for the general public accessing medical information[39]. However, it is perhaps even more pertinent for infant conditions such as GOR/GORD, where management is not clear cut and many parents seek out possible solutions to improve signs and symptoms.

The use of qualitative methodology is a key strength of this study, allowing in depth exploration of a topic with limited research in this setting and where surveys have previously dominated. Triangulation through double coding of transcripts enhances the credibility of the study and provides assurance that thematic analysis represents participants' voices[26]. However, several limitations are also acknowledged. The sample size is smaller than planned as, despite pursuing several avenues of recruitment, responses were limited. Whilst data saturation was reached for the most part, only one father was interviewed, and the study appeared to attract more parents of children with GORD rather than GOR. As a result of these limitations, study findings might lack transferability to other individuals or settings[26].

Conclusions

In conclusion, infant GOR/GORD can affect infants and parents in a variety of ways, impacting both physical and mental health. GOR/GORD is complex to manage, with difficulties administering medications that have varied effectiveness, and symptoms that overlap 'normal' infant behaviour. Parents play a vital role in management, but there is lack of consistency of information, and levels of knowledge about GOR/GORD among healthcare

professionals vary. More education on the diagnosis and management of reflux could be beneficial for parents-to-be, parents and clinicians, and further research is needed into healthcare professionals' knowledge and perceptions.

More teaching could be introduced to the training curriculum of healthcare staff, including awareness of the parental impact and the importance of managing expectations. Additionally, it could be beneficial to develop a clearer definition of GOR and its distinction from GORD, with clear evidence-based diagnostic criteria and management guidance.

Qualitative studies with fathers, to explicitly explore their experiences of infant reflux, and with health professionals (including General Practitioners, health visitors and pharmacists) to explore their knowledge and perceptions of infant GOR would help to increase understanding.

References

1. Kwok TC, Ojha S, Dorling J. Feed thickeners in gastro-oesophageal reflux in infants. BMJ Paediatr Open. 2018;2: e000262. doi:10.1136/bmjpo-2018-000262
2. National Institute for Health and Care Excellence. NICE Guideline NG1: Gastro-oesophageal reflux disease in children and young people: diagnosis and management. [cited 13 Nov 2023]. Available: https://www.nice.org.uk/guidance/ng1/ifp/chapter/reflux-in-babies
3. Great Ormond Street Hospital. Gastro-oesophageal reflux. [cited 13 Nov 2023]. Available: https://www.gosh.nhs.uk/conditions-and-treatments/conditions-we-treat/gastro-oesophageal-reflux/
4. Rosen R, Vandenplas Y, Singendonk M, Cabana M, DiLorenzo C, Gottrand F, et al. Pediatric Gastroesophageal Reflux Clinical Practice Guidelines: Joint Recommendations of the North American Society for Pediatric Gastroenterology, Hepatology, and Nutrition and the European Society for Pediatric Gastroenterology, Hepatology, and Nutrition. J Pediatr Gastroenterol Nutr. 2018;66: 516–554. doi:10.1097/MPG.0000000000001889
5. Lopez RN, Lemberg DA. Gastro-oesophageal reflux disease in infancy: a review based on international guidelines. Med J Aust. 2020;212: 40–44. doi:10.5694/mja2.50447
6. Dahlen HG, Foster JP, Psaila K, Spence K, Badawi N, Fowler C, et al. Gastro-oesophageal reflux: a mixed methods study of infants admitted to hospital in the first 12 months following birth in NSW (2000-2011). BMC Pediatr. 2018;18: 30. doi:10.1186/s12887-018-0999-9

7. McGann J, Manohar J, Hiscock H, O'Connor D, Hodgson J, Babl F, et al. Caring for crying babies: A mixed-methods study to understand factors influencing nurses' and doctors' management of infant colic. J Paediatr Child Health. 2018;54: 653–660. doi:10.1111/jpc.13858

8. Durand C, Kane C, McIlroy H, Verhoeff F. 1493 Infants attending the paediatric emergency department with feeding difficulties & referrals to nurse led infant feeding clinic. Abstracts. BMJ Publishing Group Ltd and Royal College of Paediatrics and Child Health; 2021. p. A392.2-A393. doi:10.1136/archdischild-2021-rcpch.682

9. Bell JC, Schneuer FJ, Harrison C, Trevena L, Hiscock H, Elshaug AG, et al. Acid suppressants for managing gastro-oesophageal reflux and gastro-oesophageal reflux disease in infants: a national survey. Arch Dis Child. 2018;103: 660–664. doi:10.1136/archdischild-2017-314161

10. Scherer LD, Zikmund-Fisher BJ, Fagerlin A, Tarini BA. Influence of "GERD" label on parents' decision to medicate infants. Pediatrics. 2013;131: 839–845. doi:10.1542/peds.2012-3070

11. Diaz DM, Winter HS, Colletti RB, Ferry GD, Rudolph CD, Czinn SJ, et al. Knowledge, attitudes and practice styles of North American pediatricians regarding gastroesophageal reflux disease. J Pediatr Gastroenterol Nutr. 2007;45: 56–64. doi:10.1097/MPG.0b013e318054b0dd

12. Kirby CN, Segal AY, Hinds R, Jones KM, Piterman L. Infant gastro-oesophageal reflux disease (GORD): Australian GP attitudes and practices. J Paediatr Child Health. 2016;52: 47–53. doi:10.1111/jpc.12983

13. Hodge S, Perkins E. Communicative Rationality in the Clinic? Exploring the Parental Role in the Management of Gastro-Oesophageal Reflux in Children. Soc Theory Health. 2007;5: 107–125. doi:10.1057/palgrave.sth.8700093

14. Kvello M, Åvitsland TL, Knatten CK, Fyhn TJ, Malt U, Emblem R, et al. Psychologic Distress and Anxiety in Mothers of Children With Gastroesophageal Reflux Undergoing Antireflux Surgery. J Pediatr Gastroenterol Nutr. 2019;68: 818–823. doi:10.1097/MPG.0000000000002286

15. Neu M, Corwin E, Lareau SC, Marcheggiani-Howard C. A review of nonsurgical treatment for the symptom of irritability in infants with GERD. J Spec Pediatr Nurs JSPN. 2012;17: 177–192. doi:10.1111/j.1744-6155.2011.00310.x

16. Neu M, Schmiege SJ, Pan Z, Fehringer K, Workman R, Marcheggianni-Howard C, et al. Interactions During Feeding with Mothers and Their Infants with Symptoms of Gastroesophageal Reflux. J Altern Complement Med. 2014;20: 493–499. doi:10.1089/acm.2013.0223

17. Petty J, Jarvis J, Thomas R. Understanding parents' emotional experiences for neonatal education: A narrative, interpretive approach. J Clin Nurs. 2019;28: 1911–1924. doi:10.1111/jocn.14807

18. Burr V. An Introduction to Social Constructionism. 0 ed. Routledge; 2006. doi:10.4324/9780203133026

19. Crotty MJ. The foundations of social research: meaning and perspective in the research process. Place of publication not identified: SAGE Publications Ltd; 2015.

20. Schwandt T. Three epistemological stances for qualitative inquiry: Interpretivism, hermeneutics, and social constructionism. In Denzin NK, Lincoln YS, editors, Handbook of Qualitative Research, 2 ed. SAGE Publishing; 2000. pp. 189–213.

21. Braun V, Clarke V. Using thematic analysis in psychology. Qual Res Psychol. 2006;3: 77–101. doi:10.1191/1478088706qp063oa

22. Flick U. Designing qualitative research. 2nd edition. Los Angeles London New Dehli Singapore Washington DC Melbourne: SAGE; 2018.

23. Lumivero. NVivo (Version 14). 2017. Available: www.lumivero.com

24. Williams M, Moser T. The Art of Coding and Thematic Exploration in Qualitative Research. Int Manag Rev. 2019;15.

25. Denzin NK, Lincoln YS, editors. The SAGE handbook of qualitative research. Fifth edition. Los Angeles London New Delhi Singapore Washington DC Melbourne: SAGE; 2018.

26. Patton MQ. Enhancing the quality and credibility of qualitative analysis. Health Serv Res. 1999;34: 1189–1208.

27. Miller-Loncar C, Bigsby R, High P, Wallach M, Lester B. Infant colic and feeding difficulties. Arch Dis Child. 2004;89: 908–912. doi:10.1136/adc.2003.033233

28. Petzoldt J. Systematic review on maternal depression versus anxiety in relation to excessive infant crying: it is all about the timing. Arch Womens Ment Health. 2018;21: 15–30. doi:10.1007/s00737-017-0771-4

29. Aizlewood EG, Jones FW, Whatmough RM. Paediatric gastroesophageal reflux disease and parental mental health: Prevalence and predictors. Clin Child Psychol Psychiatry. 2023;28: 1024–1037. doi:10.1177/13591045231164866

30. Hairston IS, Handelzalts JE, Lehman-Inbar T, Kovo M. Mother-infant bonding is not associated with feeding type: a community study sample. BMC Pregnancy Childbirth. 2019;19: 125. doi:10.1186/s12884-019-2264-0

31. Kennell JH, Klaus MH. Mother-infant bonding: Weighing the evidence. Dev Rev. 1984;4: 275–282. doi:10.1016/S0273-2297(84)80008-8

32. Bicking Kinsey C, Hupcey JE. State of the science of maternal-infant bonding: a principle-based concept analysis. Midwifery. 2013;29: 1314–1320. doi:10.1016/j.midw.2012.12.019

33. NHS England. Perinatal mental health. [cited 14 Nov 2023]. Available: https://www.england.nhs.uk/mental-health/perinatal/

34. Abdul Hamid HB, Szatkowski L, Budge H, Ojha S. Anti-reflux medication use in preterm infants. Pediatr Res. 2022;92: 520–525. doi:10.1038/s41390-021-01821-y

35. Leung AK, Hon KL. Gastroesophageal reflux in children: an updated review. Drugs Context. 2019;8: 212591. doi:10.7573/dic.212591

36. Tighe MP, Andrews E, Liddicoat I, Afzal NA, Hayen A, Beattie RM. Pharmacological treatment of gastro-oesophageal reflux in children. Cochrane Database Syst Rev. 2023;8: CD008550. doi:10.1002/14651858.CD008550.pub3

37. Schuetz Haemmerli N, Stoffel L, Schmitt K-U, Khan J, Humpl T, Nelle M, et al. Enhancing Parents’ Well-Being after Preterm Birth-A Qualitative Evaluation of the “Transition to Home” Model of Care. Int J Environ Res Public Health. 2022;19: 4309. doi:10.3390/ijerph19074309

38. Fairless HE, Ulloa M, McGrath B. Continuity of care when transitioning complex preterm infants from NICU to home: Parent experiences. J Neonatal Nurs. 2021;27: 273–279. doi:10.1016/j.jnn.2020.11.009

39. Eysenbach G, Diepgen TL. Labeling and filtering of medical information on the Internet. Methods Inf Med. 1999;38: 80–88.

Contributions and acknowledgments

KM, MB, LS and SO conceived the study idea and design. KM, MB and LS designed the participant information sheet, consent form and interview guide. KM recruited, consented and interviewed participants. KM facilitated transcription and quality checked each transcript. KM coded each transcript, ST coded a sample of transcripts for triangulation and MB also reviewed two transcripts to help ensure the themes reflected the data. KM conducted the thematic analysis, with comment on themes from MB and LS. KM wrote a first draft of the manuscript. All authors edited and approved the final version.

Semi-structured interview guide

Click here to access/download
Supporting Information
McVicar et al. Supporting information file S1.docx

Confirmation of ethical approval

Click here to access/download
Supporting Information
McVicar et al. Confirmation of ethical approval.pdf
